# Characterization of Exosomes and Exosomal RNAs Isolated from Post-Mortem Body Fluids for Molecular Forensic Diagnosis

**DOI:** 10.3390/diagnostics12092153

**Published:** 2022-09-05

**Authors:** So-Yeon Kim, Sinae Jang, Sookyoung Lee, Jong-Tae Park, Su-Jin Lee, Hyung-Seok Kim

**Affiliations:** 1 Department of Forensic Medicine, Chonnam National University Medical School, Gwangju 61469, Korea; 2 Brain Korea 21 Plus Program, Chonnam National University Medical School, Gwangju 61469, Korea; 3 Division of Forensic Medical Examination, National Forensic Service, Wonju 26460, Korea; 4 Biomedical Research Institute, Chonnam National University Hospital, Gwangju 61469, Korea

**Keywords:** exosome, post-mortem, body fluid, exosomal RNA

## Abstract

Exosomes have been mainly studied for their potential applications in biomarker detection and drug delivery for diagnosis and treatment. However, in the field of forensic research, the potential value of exosomes derived from post-mortem body fluids has not been investigated to date. Here, we isolated the exosomes and exosomal RNAs from post-mortem body fluids, including cardiac blood, pericardial fluid, and urine. We also compared commercial exosome isolation kits to determine the optimal method for post-mortem exosome isolation. Transmission electron microscopy (TEM), the Agilent bioanalyzer system, and western blotting were used to evaluate the efficiencies of alternative isolation methods and the characteristics of isolated exosomes. There were no significant differences between exosomes obtained from post-mortem and ante-mortem body fluids in the expression of exosome surface markers or morphology. The exosomes were well-preserved even under simulated post-mortem conditions. Among the isolation procedures tested, the membrane affinity column-based method was the most suitable for post-mortem exosomal RNA isolation. These results suggest that exosomes are well-preserved in post-mortem body fluids and could be utilized for forensic diagnosis.

## 1. Introduction

Post-mortem diagnosis of myocardial infarction (MI) requires careful assessment using a comprehensive clinicopathological approach [1]. Biomarkers that allow post-mortem examination are needed for accurate diagnosis of the cause of death. It is a challenge to adopt clinical diagnostic markers for forensic use because bioactive materials, such as DNA, RNA, and proteins, are rapidly degraded after death [2,3]. Recently, other biomarkers, including small non-coding RNAs, have been proposed for use in forensic investigations. The high levels of microRNAs available in blood and other body fluids help reveal valuable information; this is because pathophysiological conditions and tissue damage are well reflected in the stability and specificity of microRNAs. Overall, these features make microRNAs ideal candidates for circulating biomarkers in forensic bioanalytical procedures (PMID:26554231). However, considering the post-mortem changes in the body, more stable biomarkers are needed.

Exosomes are extracellular vesicles of approximately 30–150 nm. They are released from the cell via the exocytotic pathway [4,5]. They act as excellent delivery vehicles in the body; exosomes carry their cargo, such as proteins, DNA, and RNA, contained in a bilayer membrane protecting them from enzymatic degradation and deliver them to target cells [6,7]. Exosomes can be detected in all body fluids, such as the cerebrospinal fluid, saliva, breast milk, blood, and urine; in addition, they can provide biological information on health conditions [8,9]. Extracellular vesicles including exosomes are widely used as biomarkers of cardiovascular disease to assess disease pathogenesis, severity, progression, and therapeutic modality [10,11]. However, no studies are available on the use of exosomes originating from body fluids for post-mortem diagnosis.

The biochemical properties of post-mortem body fluids rapidly change; moreover, body fluid components, such as DNA, RNA, and proteins, are gradually degraded and prone to disintegration upon processing. Therefore, the method of exosome isolation is critical for accurate post-mortem analysis. The most widely used protocol for exosome isolation is differential ultracentrifugation, where exosomes are obtained through sequential centrifugation steps performed at increasing speeds [12]. However, since this method requires a large volume of liquid samples, it has limited use in the investigation of exosomes extracted from post-mortem body fluids. We compared three different types of exosome and exosomal RNA isolation kits (ExoQuick™, exoRNeasy, and ExoLute^®^) to determine the most suitable method for obtaining exosomes from post-mortem body fluids.

The aim of this study was to test whether the exosomes can be efficiently isolated from the post-mortem body fluids and whether they are preserved under conditions favoring degradation. Our findings are the first report about the characteristics of post-mortem body fluid exosomes and may help overcome the limitations of existing biomarkers in their post-mortem applications by suggesting a new potential tool for forensic diagnosis.

## 2. Materials and methods

### 2.1. Sample Collection and Preparation

Cardiac blood (n = 128), pericardial fluid (n = 143), and urine (n = 5) samples were consecutively obtained from autopsies conducted from 2020 to 2022 at Chonnam National University. Among them, samples with suspected post-mortem intervals (PMIs) within 48 h were used in this study. For control, whole blood samples collected over same period from healthy donors were provided by the hematopoietic stem cell transplant center at Chonnam National University Hwasun Hospital. The collection and use of these samples for this study were approved by the National Forensic Service Institutional Review Boards (permission #: 906-220421-BR-008-02). Cardiac blood samples were transported in vacutainer tubes containing EDTA. Pericardial fluid samples were transported in 15 mL conical tubes. On the day of collection, plasma was separated from cardiac blood through repetitive centrifugation at 1550× *g* for 30 min and at 3200× *g* for 30 min to remove the cells [13]. Plasma and pericardial fluid samples were filtered using 0.45 μm syringe filters. Aliquoted samples were stored at –80 °C to be used in subsequent experiments. 

### 2.2. Simulation of Post-Mortem Changes in Blood Samples

Whole blood samples from healthy donors (n = 10) were placed on a shaker running at 35 rpm at room temperature for 48 h to mimic post-mortem conditions. Plasma was separated and collected after 0, 24, and 48 h to observe whether the exosomes were well-preserved in an environment favoring degradation, similar to that of a dead body.

### 2.3. Exosome and Exosomal RNA Isolation and Quantification

Exosomes from 250 μL plasma obtained from dead bodies (n = 8) were isolated using an ExoQuick™ kit (System Biosciences Inc., Palo Alto, CA, USA) and an ExoLutE^®^ exosome isolation kit (Rosetta Exosome Inc., Seoul, Korea) according to the manufacturer’s instructions. Since the exosomes isolated with ExoQuick™ form a pellet, exosomal RNA was extracted using RNAiso Plus (Takara bio Inc., Shiga, Japan). Exosomes isolated using ExoLute^®^ are in liquid form; thus, exosomal RNA extraction was performed using a TRIzol^®^ LS Reagent (InVitrogen, Carlsbad, CA, USA) according to the manufacturer’s instructions. To compare the extracts obtained using the two kits, we used an exoRNeasy midi kit (Qiagen, Hilden, Germany); this allows exosomal RNA extraction in a single step. In brief, pre-filtered plasma and pericardial fluid were mixed with a binding buffer (XBP) and added to the membrane affinity column. After discarding the flow-through using a centrifuge, a washing buffer (XWP) was added to the column; and the exosomes were mixed with a lysis reagent (QIAzol^®^) passing through the membrane and extracted to the bottom of the tube. Then, chloroform was added to remove the phenol component; and the clear layer containing the isolated RNA was separated through centrifugation. After several washing steps, exosomal RNAs were finally obtained. The purity and quantity of the isolated exosomal RNA were measured using a Nanodrop™ 2000 spectrophotometer (ThermoFisher Scientific, Wilmington, DE). The RNA quality was assessed using the Agilent 2100 bioanalyzer instrument with the RNA 6000 Pico Chip (Agilent Technologies, Amstelveen, The Netherlands) according to the manufacturer’s protocol.

### 2.4. Western Blot Analysis

The exosomes were isolated using the ExoQuick™ kit (System Biosciences Inc., Palo Alto, CA, USA) from plasma samples from healthy donors (n = 3), plasma samples from dead bodies (n = 3), and pericardial fluid samples from dead bodies (n = 3). The isolated exosomes were then lysed in a PRO-PREP protein extraction solution (iNtRON Biotechnolog, Seongnam, Korea). Soluble protein extracts containing 10 µg protein per sample were loaded into sodium dodecyl sulfate polyacrylamide gels and transferred onto nitrocellulose membranes (PALL, Port Washington, NY, USA) by electrophoresis. Membranes were incubated in 5% non-fat dry milk in Tris-buffered saline with 0.2% Tween 20 for blocking; followed by overnight incubation at 4 °C with antibodies for the exosome surface markers CD9 (1:600, Proteintech, 25682-1-AP) and CD63 (1:300, Proteintech, 20597-1-AP). After incubation with secondary antibodies, the membranes were visualized using a chemiluminescence reagent containing a luminal reagent and peroxide solution (Merck Millipore, Darmstadt, Germany). Optical densities were measured by a ChemiDoc XRS+ System (Bio-Rad, Hercules, CA, USA). Signal intensities were quantified using ImageJ software (National Institute of Mental Health).

### 2.5. Transmission Electron Microscopy (TEM)

The exosomes were isolated using the ExoQuick™ kit (System Biosciences Inc., Palo Alto, CA, USA) from plasma samples from healthy donors (n = 2), plasma samples from dead bodies (n = 2), and pericardial fluid samples from dead bodies (n = 4). The isolated exosomes were immediately resuspended in 50–100 µL of phosphate buffered saline (PBS) depending on the size of the exosome pellets. The samples were fixed with a mixture of 2% glutaraldehyde (*v*/*v*) and 2% paraformaldehyde (*v*/*v*) in a 0.05 M cacodylate buffer (pH = 7.2) at room temperature for 4 h. The fixed samples were washed with the same buffer; and the isolated exosomes (2.5 µL) were dried on freshly glow discharged 150 mesh carbon-coated TEM grids, negatively stained with 2% aqueous uranyl acetate, and observed using a JEM-2400F TEM (JEOL Ltd., Akishima, Japan) operated at 200 kV. The images were captured with a bottom mounted 4K Rio-16 CMOS type digital camera (Gatan Inc., Pleasanton, CA, USA).

### 2.6. Statistical Analysis

All the experimental data were presented as the mean ± SEM (standard errors of the mean), and the expression level of 0 h was used for normalization. The Kruskal-Wallis one-way ANOVA followed by Dunn’s post hoc test were performed for comparisons between the groups. GraphPad Prism 8.0.1 (GraphPad Software Inc., San Diego, CA, USA) was used to process the data, and *p* values < 0.05 were considered statistically significant.

## 3. Results

### 3.1. Identification and Evaluation of Exosomes Isolated from Post-Mortem Body Fluids

**Figure 1:**
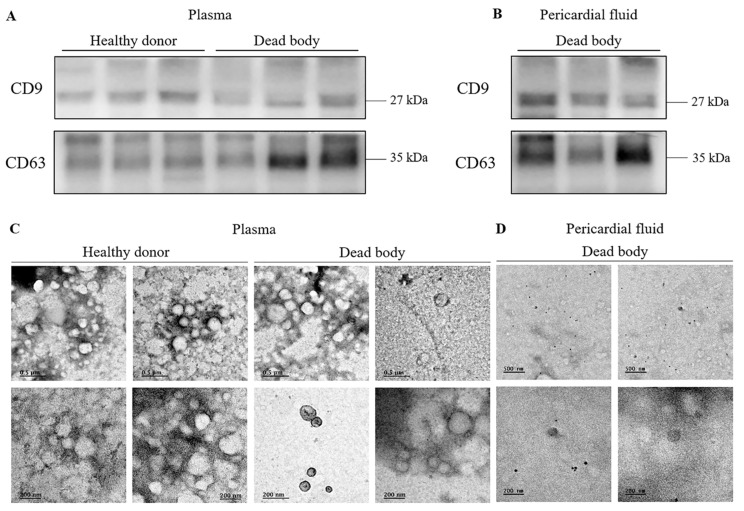
Analysis of the exosome derived from the post-mortem body fluids. (**A**) Western blot performed on three different samples of exosomes isolated from the plasma of healthy donors (n = 3) and dead bodies (n = 3), and (**B**) the pericardial fluid of dead bodies (n = 3) to detect exosome surface proteins (CD9, CD63). (**C**) Transmission electron microscopy images of exosomes derived from the plasma of healthy donors and dead bodies, and from (**D**) the pericardial fluid of dead bodies. The scale bar = 200 nm or 500 nm (0.5 µm).

In this study, to evaluate the basic characteristics of body fluid exosomes, we separately isolated exosomes from the plasma, pericardial fluid, and urine obtained post-mortem using different exosome extraction kits: ExoQuick and ExoLute. Purity tests revealed that albumin contamination was present in serum rather than in plasma, in contrast to a previous report [14]. Therefore, we used the plasma in subsequent experiments. Although the size and color of the pellets differed among samples, we confirmed that exosome pellets were successfully obtained at the bottom of the tube. Exosome surface protein expression was confirmed by western blot analysis to verify proper exosome isolation. CD9 [15] and CD63 [16] were detected in the exosomes isolated from the plasma of healthy donors and dead bodies, as well as from the pericardial fluid of dead bodies. There was no difference in expression levels between the exosomes obtained from healthy donors and dead bodies (Figure 1A,B).

Next, we visualized the exosomes using transmission electron microscopy (TEM) to examine their morphology. ‘Cup-shaped’ particles, one of the typical characteristics of exosomes [17], were observed in the dead body samples as well as in samples from healthy donors. The diameters of the majority of the particles isolated with the three methods were approximately 30–150 nm, consistent with previously reported exosome size distributions. There was no significant difference in morphology between the exosomes obtained with the two different isolation kits or those obtained from the different sample types (Figure 1C,D).

### 3.2. Sustained Expression of Exosome Surface Markers in Exosomes Derived from Plasma of Healthy Donors

**Figure 2:**
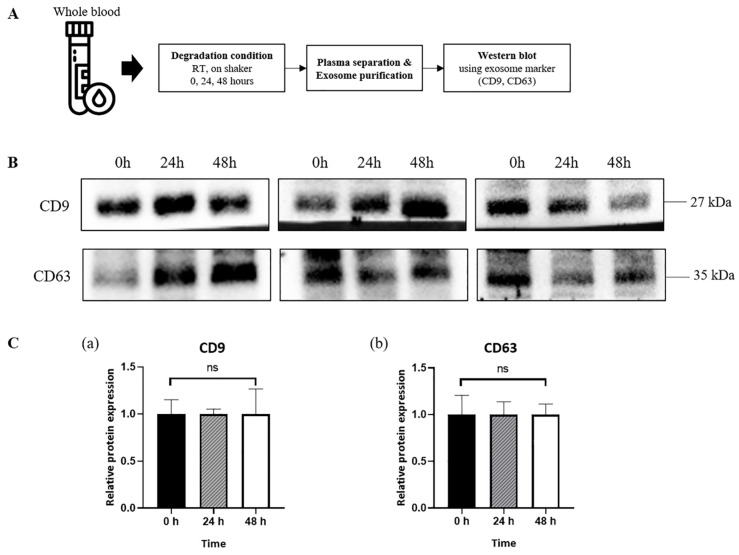
Sustained expression of the exosome surface proteins in the plasma exosomes of the healthy donors. (**A**) A description of the entire experimental process. (**B**) The western blot result of the healthy donors (n = 3) for the exosome surface proteins at 0, 24, and 48 h after isolation. (**C**) The relative protein expression of CD9 (**a**) and CD63 (**b**) were presented as the mean ± standard error of the mean (SEM) of the three experiments. Ns: not significant.

Post-mortem blood analysis was performed for forensic diagnosis after a 24–48-h delay following death. Therefore, it was necessary to evaluate the sequential changes in the exosomes and surface marker expression to compare the samples obtained post-mortem and those obtained from healthy donors. The expression of CD9 and CD63 was observed through western blot after the isolation of exosomes from the plasma of healthy donors, 0, 24, and 48 h after isolation (Figure 2A). Furthermore, there was no statistical significance in CD9 and CD63 expression levels over time (Figure 2B,C).

### 3.3. Bioanalyzer Electropherogram Analysis of Exosomal RNA Derived from Plasma from Dead Bodies and Healthy Donors

**Figure 3:**
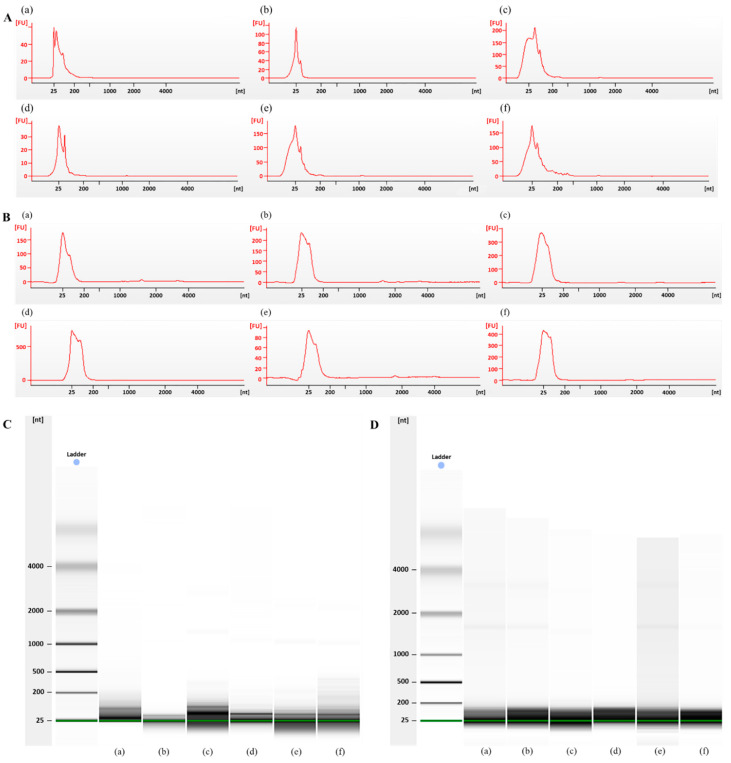
Evaluation of the exosomal RNA quality in the plasma of healthy donors and dead bodies. (**A**) Electrophoretic spectrums and (**C**) gel images of the exosomal RNA species derived from the plasma of healthy donors at 0 (**a**,**d**), 24 (**b**,**e**), and 48 (**c**,**f**) h after isolation. (**B**) Electrophoretic spectrums and (**D**) gel images of the exosomal RNA from the plasma of dead bodies from six different autopsy cases (**a**–**f**). FU, fluorescence units; nt, nucleotides.

Nucleic acids, including those found in exosomes in body fluids, constitute a highly stable type of disease biomarkers (PMID:25684126). The analysis of RNA molecules, especially some specific microRNAs, as forensic biomarkers has already been described; and is widely used for determining the cause of death in cases of myocardial infarction [18], drowning [19], or traumatic brain injury [20]. However, for forensic autopsies, samples are obtained from at least partly putrefied dead bodies; such conditions raise the question of whether forensic RNA research can provide any valuable information at all. So, we also tested for post-mortem body fluid samples and living donor blood samples for RNA purity with a time-dependent series. We compared the peak patterns and migration patterns after the isolation of exosomal RNA from the plasma of healthy donors and dead bodies using an exoRNeasy isolation kit (Qiagen, Hilden, Germany). The quantity and quality of the exosomal RNAs are shown in Table 1. In both groups, a peak for small RNAs (25–200 nt) was detected; however, no peak was found for the ribosomal RNA (Figure 3A,B). The RNA smears of all the samples in the gel lay appear to be in the range of 25–200 nucleotides; this is indicative of small RNAs (Figure 3C,D). Some cases showed two very faint bands, representing the 28S and 18S rRNA. These results suggest that the exosomes isolated from post-mortem blood mainly contain small RNAs and could be used for molecular forensic diagnosis.

### 3.4. Comparison of Exosome Isolation Kits for Exosomal RNA Extraction from Post-Mortem Body Fluids

Exosome isolation yields from body fluids greatly varied according to the method of isolation. In addition, considering the post-mortem biochemical changes and limited sample volumes, it is necessary to determine the most reliable method for post-mortem body fluid exosome isolation. In this study, among the encapsulating exosome contents, we compared the concentration (ng/μL) of the exosomal RNA isolated from post-mortem plasma among samples prepared with three different kits: ExoQuick™ (System Biosciences Inc., CA, USA), exoRNeasy (Qiagen, Hilden, Germany), and ExoLute^®^ (Rosetta Exosome Inc., Seoul, Korea). As shown in Table 2, there were significant differences in the RNA yield among the kits tested. ExoQuick™ generated the highest exosome yields; and the RNA yield was the highest on average with exoRNeasy, followed by ExoQuick™. Compared with the other kits, the concentration of RNA obtained using ExoLute^®^ was drastically low in case of RNA isolation from the body fluids originating from dead bodies (Table 1).

## 4. Discussion

Post-mortem biochemical analyses can be a promising resource for forensic pathologists to diagnose the cause of death. However, it takes about two days on average to perform an autopsy; moreover, biomolecules are rapidly degraded after death. Therefore, there are limitations for the application of post-mortem biomarker tests that are widely used in clinical medicine [21]. Although exosomes can provide pathobiological information as clinical biomarkers, there have been only a few reports on exosome analysis as a diagnostic tool in forensic medicines. Herein, we assessed the potential use of exosomes, which are more stably preserved after death and contain pathological information, as a new biomarker for post-mortem diagnosis.

We found that exosomes were successfully isolated from post-mortem blood and pericardial fluid, and their morphology was not much different from that of ante-mortem exosomes. In addition, the isolated exosomes and encapsulated small RNAs, which were 20–200 nucleotide long, were stably preserved under simulated degradation conditions. These suggest that exosomes can overcome the limitations of existing biomarkers. In order to identify the optimal exosome isolation method to obtain the highest yield, we compared three different types of exosome isolation kits; we found that the method using a membrane affinity column is the most suitable for the post-mortem isolation of exosomal RNA from body fluids.

Although the molecular mechanisms regulating exosome secretion have not been clearly understood, cellular stress such as disease-associated hypoxia is known to increase exosome release [22]. Since microRNAs, which account for most of the RNAs in exosomes [23], regulate the function in target cells in a disease-specific manner, they have been investigated as biomarkers for various diseases, such as acute myocardial infarction and cancer, among others [24]. While the post-mortem stability of RNA varies by RNA type, it is known that small non-coding RNAs are more stable under post-mortem conditions than other molecules [25]. We compared the yield and stability of exosomal RNAs using different types of exosome isolation methods in various post-mortem body fluids.

As the first step of exosome isolation, several methods of extracting pure exosomes are still being actively studied. Ultracentrifugation is the classical and, accordingly, the most widely used method [26]. This method, however, is not suitable for isolating exosomes from post-mortem body fluids; this is because the amount of body fluids obtained from autopsy is not sufficient for the procedure. Instead, we used three commercial kits for extraction of exosomal RNA: ExoQuick™ (System Biosciences Inc., Palo Alto, CA, USA), exoRNeasy (Qiagen, Hilden, Germany), and ExoLute^®^ (Rosetta Exosome Inc., Seoul, Korea).

ExoQuick™ works on the principle of the precipitation of exosomes into a pellet using a polymer-based solution. Even though the method has the advantage of taking less time, some false positives can be generated as other particles can precipitate together with the exosomes. ExoLute^®^ is a method that removes various contaminants; these are likely to precipitate together with EVs, by spin-exclusion chromatography following the precipitating and re-solubilization of exosomes. However, it is difficult to refine exosomes as spin-sec, the second step, does not work favorably when there are large amounts of EVs or aggregates. In addition, in this experiment, the concentration and purity of exosomal RNAs extracted with the ExoLute^®^ kit were much lower compared to those obtained with the other two kits. Although not much is known about biochemical changes taking place in blood after death [27], we believe that large amounts of EVs and other aggregates in post-mortem body fluids are agglomerated during the precipitation step; thus, their passage through the spin-sec was difficult. On the other hand, exoRNeasy captures exosomes from body fluids using a membrane affinity spin column; the captured exosomes are lysed on the column. Then, exosomal RNA can be directly extracted from the Qiagen RNeasy MinElute column. This method is the most appropriate one for the additional analysis of exosomal RNA extracted from post-mortem body fluids; this is because the exosomes are well-extracted from the column without any additional steps. Hence, they are less likely to be lost or contaminated, compared with the other methods based on precipitation and spin-sec.

Exosomes have also been extracted from urine in previous clinical research. Therefore, we also attempted to analyze exosomes derived from post-mortem urine samples. However, exosome isolation form dead body urine was inadequate for forensic research; this is due to the insufficient amount of urine found in the urinary bladder of a dead body. In addition, since most substances are filtered by the kidney (except in the cases of certain disease conditions), it is thought that there are very low amounts of exosomes present in the urine. Consistent with this idea, the RNA purity and yield were extremely low in the urine samples (data not shown). Thus, for a proper analysis of urinary exosomes, we suggest performing ultracentrifugation starting with a large volume of a sample of more than 50 mL [28].

To our knowledge, there have been no studies on exosomes derived from post-mortem body fluids. Therefore, this study, for the first time, introduces the possibility that exosomes can be utilized not only in clinical, but also in forensic studies. The presence and stability of exosomes in post-mortem specimens and an appropriate extraction method were confirmed. By conducting research to find disease-specific miRNAs in exosomes, it is thought that it could be helpful for clinical diagnosis and determining cause of death. 

## 5. Conclusions

In this study, we confirmed that exosomes can be successfully isolated from post-mortem body fluids. The post-mortem exosomes did not significantly differ in morphology from ante-mortem exosomes and were well-preserved in simulated degradation conditions. This suggests the possibility that exosomes can be used as biomarkers in the field of forensic science. In addition, we found that the membrane affinity spin column-based methods were most suitable for the isolation of exosomal RNA with high yields from post-mortem body fluids. We expect that this study can be used as a basis for the analysis of exosomes derived from post-mortem body fluids in the future.

## Figures and Tables

**Table 1:** Quantity and quality of the exosomal RNA using the exoRNeasy isolation kit.

Group	Sample	Concentration (ng/µL)	OD 260/280
Healthy donors (Figure 3A,C)	(a)	19.5	1.68
	(b)	32.8	2.07
	(c)	48.8	1.91
	(d)	382.3	1.99
	(e)	10.5	1.89
	(f)	91.3	2.03
Dead bodies (Figure 3B,D)	(a)	9.1	1.42
	(b)	19.8	1.82
	(c)	129.1	2.02
	(d)	25.7	1.68
	(e)	120.3	2.02
	(f)	407.5	2.02

**Table 2:** Comparison of the exosomal RNA yield according to the exosome extraction kit used.

Kit (End Product)	Company	Basic Concept	Plasma Volume, μL	Time, min	Complexity	Concentration, ng/μL	Mean ± SD
ExoQuick™ (exosome)	System biosciences	Precipitation (polymer)	250	65	Easy	134.3	151.1 ± 100.7
						91.1	
						117.9	
						86.5	
						253.6	
						354.3	
						58.6	
						112.4	
exoRNeasy (exosomal RNA)	Qiagen	Membrane affinity	250	55	Easy	112.8	159.4 ± 119.2
						98.2	
						117.6	
						48.7	
						370.8	
						316.7	
						58.9	
						151.7	
ExoLute^®^ (exosome)	Rosetta exosome	Precipitation (polymer) and size exclusion chromatography	250	89	Easy	3.2	3.9 ± 1.4
						1.8	
						3.0	
						6.3	
						4.5	
						3.8	
						3.5	
						5.3	

## Data Availability

All relevant data are within the manuscript.
